# Do the equity-efficiency preferences of the Israeli Basket Committee match those of Israeli health policy makers?

**DOI:** 10.1186/s13584-017-0145-4

**Published:** 2017-04-30

**Authors:** Amir Shmueli

**Affiliations:** The Hebrew University-Hadassah School of Public Health, Jerusalem, Israel

**Keywords:** Efficiency, Equity, Policymakers, Health, DCE, Basket Committee, Prioritization

## Abstract

**Background:**

Prioritization of medical technologies requires a multi-dimensional view. Often, conflicting equity and efficiency criteria should be reconciled. The most dramatic manifestation of such conflict is in the prioritization of new medical technologies asking for public finance performed yearly by the Israeli Basket Committee. The aim of this paper is to compare the revealed preferences of the 2006/7 Basket Committee’s members with the declared preferences of health policy-makers in Israel.

**Methods:**

We compared the ranking of a sample of 18 accepted and 16 rejected technologies evaluated by the 2006/7 Basket Committee with the ranking of these technologies as predicted based on the preferences of Israeli health policy-makers. These preferences were elicited by a recent Discrete Choice Experiment (DCE) which estimated the relative weights of four equity and three efficiency criteria. The candidate technologies were characterized by these seven criteria, and their ranking was determined. A third comparative ranking of these technologies was the efficiency ranking, which is based on international data on cost per QALY gained.

**Results:**

The Committee’s ranking of all technologies show no correspondence with the policy-makers’ ranking. The correlation between the two is negative when only accepted technologies are ranked. The Committee’s ranking is positively correlated with the efficiency ranking, while the health policy-makers’ ranking is not.

**Discussion:**

The Committee appeared to assign to efficiency considerations a higher weight than assigned by health policy-makers. The main explanation is that while policy-makers’ ranking is based on stated preferences, that of the Committee reflects revealed preferences. Real life prioritization, made under a budget constraint, enhances the importance of efficiency considerations at the expense of equity ones.

**Conclusions:**

In order for Israeli health policy to be consistent and well coordinated across policy-makers, some discussions and exchanges are needed, to arrive at a common set of preferences with respect to equity and efficiency considerations.

## Background

There is a traditional and long-standing tension in economics and public policy between efficiency - defined as the maximization of welfare - and equity, which includes considerations of equality, the distribution of welfare and social justice.

In terms of health policy, the aspiration to efficiency is equivalent to the maximization of health. When health is measured as quality-adjusted life years (QALYs), as is the case in economic assessments of health technologies, efficiency is identified with the maximization of QALYs. However, maximization of health itself does not take into account considerations of equality, justice, medical need, etc. [1]. On the other hand, when health policy is concerned with the needy or poor, some efficiency might have to be given up.

Policy-makers try to reconcile between efficiency and equity considerations when formulating health policy [2]. Such conciliation is particularly difficult in the prioritization of new medical technologies facing a limited budget, as is the case of the decisions made by the members of the Israeli Basket Committee.

While general guidelines and principles were offered to the members of the Committee [3], their actual decisions were never analysed with respect to the conciliation of equity and efficiency criteria.

The 2006/7 Basket Committee reported not only its inclusion decisions, but also ranked the accepted technologies. An earlier study [4] examined the concordance between this prioritization of technologies and prioritization based on international data on cost per QALY gained by these technologies, the most significant efficiency criterion. The findings indicated that while the concordance is weak and several marked differences exist, the correlation between the two rankings is significantly positive, and in general, the inclusion decisions – as well as the prioritization of the accepted technologies - might be justified on efficiency grounds.

A recent Discrete Choice Experiment (DCE) elicited the declared preferences of Israeli health policy-makers with regard to efficiency and equity considerations. The results indicated that the preferences of Israeli health policy-makers with regard to equity and efficiency criteria are quite balanced. This balance is quite exceptional when comparing the Israeli preferences to those declared by health policy-makers in other countries [5].

### Objectives

The objective of this study is to compare the revealed ranking of the technologies evaluated by the 2006/7 Basket Committee’s members with the ranking derived from the declared preferences of health policy-makers in Israel.

## Methods

A recent DCE estimated the relative weights of equity and efficiency criteria in the preferences of Israeli health policy-makers [5].

The criteria were taken from an international study [6], and were adapted to the Israeli reality. Four equity criteria were used: severity of the disease (measured as less or more than 2 healthy years), age of treated patients (young vs. others and elderly vs. others), and the extent to which the subsidization of the poor is important. The three efficiency criteria included the potential number of beneficiaries (more or less than 100,000), the extent of the health benefits to the patient (less or more than 5 healthy years), and the results of economic assessments (cost per QALY gained is less or more than GNP per capita).

Since national health insurance systems, such as the Israeli one, are based on solidarity and cross-subsidization between the rich/ healthy and the sick/ poor, the criterion relating to the “subsidization of the poor” was reformulated as “the technology should be financed publicly so that the poor could afford it” (yes or no). The operational definition of affordability was based on actual policy: the monthly copayment ceiling for chronic patients was about 250 NIS in 2006, and we assumed that a monthly cost of a technology - if not financed publicly - of less than 125 NIS is affordable to the poor. This threshold is equivalent to 10% of the Israeli official poverty line in 2006. Consequently, technologies with a monthly cost of use of less than 125 NIS are characterized by “no” for this criterion, and more expensive technologies are characterized by “yes”. In order to explore the effect of the threshold in the criteria “the technology should be financed publicly so that the poor can use it” (125 NIS), we repeated the analysis with a threshold of 250 NIS.

The sample included past and present senior managers from the Ministry of Health, Ministry of Finance, the Sickness Funds, the Israeli Medical Associations, and hospitals’ directors. Sixty-five out of 146 policy-makers completed the survey. Due to technical problems, only 40 of them completed the demographic questions. The survey did not need a Helsinki approval.

The weights of the seven criteria were estimated using DCE methodology by the software 1000Minds. For more details see [5]. Once these weights are known, any technology, characterized by the seven criteria, can be evaluated and assigned a “desirability score”. This score indicates the likelihood that the technology – characterized by its own levels on the criteria, and using the estimated weights of the criteria – is preferred to other technologies (characterized by their own profiles of the criteria’s levels).

We focus in this paper on the decisions and prioritization made by the 2006/7 Basket Committee. In addition to deciding which technologies were included in the Basket financed by public funds, the Committee also ranked the accepted technologies.

Using the material presented to the Committee and consultations with physicians, we characterized random samples of 18 accepted technologies and 16 rejected technologies as profiles of the 7 criteria. Each technology was assigned a desirability score, predicted using the estimated weights, and the ranking of the entire set – as well as of the accepted technologies only – resulted. We derived the ranking for the entire sample of Israeli health policy-makers, as well as that for physicians and those who have ever served on the Basket Committee.

## Results

The exact definition of the seven criteria and their weights – as well as the total weight of the equity and efficiency criteria – in the selected sub-groups of health policy-makers, are presented in Table 1 (further details on the international comparison can be found in [5]).

**Table 1:** Relative weights of the equity and efficiency criteria

		Equity criteria				Efficiency criteria					
	Number of respondents	The technology is intended for patients suffering from a serious disease (life expectancy is less than 2 healthy years): Yes	The Technology is intended to treat a disease common among children: Yes	The technology is intended to treat a disease common among the elderly: Yes	Funding the technology is required so that the poor can use it: Yes	Cost per Quality Adjusted Life Year: Less than the GNP per capita	The Number of patients requiring the technology: more than 100,000	Individual Benefit: addition of more than 5 healthy years	Efficiency Weight	Equity Weight	Total
Physicians	25	11	16	9	20	12	14	18	44	56	100
Non-physicians	15	8	15	9	19	13	18	18	49	51	100
Ever served on Basket Committee	11	9	15	9	16	16	16	20	51	49	100
Never did	29	10	16	9	21	11	16	17	44	56	100
Respondents answering the demographic questionnaire	40	10	16	9	20	12	16	18	46	54	100
Respondents not answering the demographic questionnaire	25	13	11	12	17	12	13	21	47	53	100
Total	65	11	14	10	19	12	15	19	46	54	100

The two most important criteria in all sub-groups and in the entire sample are "funding the technology is necessary so that the poor will also be able to use it", clearly an equity criterion, and "benefit to the individual", a major efficiency criterion. The criteria whether the technology was intended primarily for the elderly or to treat a severe disease were the least important in all subgroups, both of them being equity criteria.

In the entire sample, the efficiency criteria have a total weight of 46% and the equity criteria – 54%. Physicians tend slightly more toward equity criteria (total weight = 56%), and those who have ever served on a Basket Committee tend more to efficiency criteria (total weight = 51%).

Tables 2 and 3 present side by side four rankings of the 34 technologies evaluated by the Committee (the technologies appear in the Tables according to their Committee’s rank), using the two monthly cost thresholds – 125 and 250 NIS respectively. These rankings include the rankings derived from the total sample of health policy-makers, the physicians and members of the past and present Basket Committees in the sample, and the efficiency ranking according to cost per QALY gained.

**Table 2:** The Committee’s and the sample ranking of technologies evaluated by the 2006/7 Basket Committee (monthly cost threshold = 125 NIS)

Technology	Committee’s inclusion decision	Rank Basket Committee	Rank survey - Total	Rank survey - Physicians	Rank survey - Members of any Basket Committee	Rank efficiency^a^
Crestor Ezetrol	YES	1	18	18	15	12
Atacand Ocsaar Diovan Olmetec	YES	2	29	29	25	3
Lantus Levemir	YES	3	29	29	25	5
Apidra Humalog Novorapid	YES	4	29	29	25	9
Eloxatin	YES	5	15	15	15	4
Xeloda	YES	6	15	15	15	1
Herceptin	YES	7	11	11	12	23
Hepsera	YES	8	3	3	3	16
Viread	YES	9	9	8	7	22
Prevnar	YES	10	33	33	33	32
Mabthera	YES	11	1	1	1	14
Keppra	YES	12	21	21	24	28
Zyprexa	YES	13	22	22	22	6
Exjade	YES	14	5	4	4	19
Plavix (cardiac)	YES	15	8	8	5	10
Growth hormon	YES	16	14	14	11	13
Plavix (stroke)	YES	17	1	1	1	8
Velcade	YES	18	4	5	5	27
Zomera	NO	26.5	11	11	12	29
Apo-Go	NO	26.5	6	6	9	21
Azilect	NO	26.5	6	6	9	17
Cetuximab (Erbitux®)	NO	26.5	25	24	25	31
Doxil	NO	26.5	15	15	15	11
Emend	NO	26.5	25	24	25	33
Erlotinib (Tarceva®)	NO	26.5	25	24	25	30
Faslodex	NO	26.5	11	11	12	24
Forteo	NO	26.5	28	28	25	34
Gardasil	NO	26.5	9	8	7	20
Lucentis	NO	26.5	18	18	15	18
Rimonabant (Acomplia®)	NO	26.5	34	34	34	25
Sifrol	NO	26.5	20	20	21	26
Spiriva	NO	26.5	29	29	25	7
Zemplar	NO	26.5	22	22	22	15
Zyban	NO	26.5	24	27	20	2
Mean rank	YES	9.5	14.8	16.9	16.1	14.0
	NO	26.5	19.3	19.2	18.8	21.4

^a^By international data on cost per QALY gained

**Table 3:** The Committee’s and the sample ranking of technologies evaluated by the 2006/7 Basket Committee (monthly cost threshold = 250 NIS)

Technology	Committee’s inclusion decision	Rank Basket Committee	Rank survey - Total	Rank survey - Physicians	Rank survey - Members of any Basket Committee	Rank efficiency^a^
Crestor Ezetrol	YES	1	28	28	25	12
Atacand Ocsaar Diovan Olmetec	YES	2	28	28	25	3
Lantus Levemir	YES	3	17	17	14	5
Apidra Humalog Novorapid	YES	4	28	28	25	9
Eloxatin	YES	5	14	14	14	4
Xeloda	YES	6	14	14	14	1
Herceptin	YES	7	10	10	11	23
Hepsera	YES	8	2	2	2	16
Viread	YES	9	9	7	7	22
Prevnar	YES	10	32	32	32	32
Mabthera	YES	11	1	1	1	14
Keppra	YES	12	20	20	24	28
Zyprexa	YES	13	21	21	22	6
Exjade	YES	14	4	4	3	19
Plavix (cardiac)	YES	15	7	7	4	10
Growth hormon	YES	16	13	13	10	13
Plavix (stroke)	YES	17	7	7	4	8
Velcade	YES	18	3	3	4	27
Zomera	NO	26.5	10	10	11	29
Apo-Go	NO	26.5	5	5	8	21
Azilect	NO	26.5	5	5	8	17
Cetuximab (Erbitux®)	NO	26.5	25	25	25	31
Doxil	NO	26.5	14	14	14	11
Emend	NO	26.5	33	33	33	33
Erlotinib (Tarceva®)	NO	26.5	25	25	25	30
Faslodex	NO	26.5	10	10	11	24
Forteo	NO	26.5	27	27	25	34
Gardasil	NO	26.5	23	23	19	20
Lucentis	NO	26.5	17	17	14	18
Rimonabant (Acomplia®)	NO	26.5	34	34	34	25
Sifrol	NO	26.5	19	19	21	26
Spiriva	NO	26.5	28	28	25	7
Zemplar	NO	26.5	21	21	22	15
Zyban	NO	26.5	23	23	19	2
Mean rank	YES	9.5	14.3	14.2	13.4	14.0
	NO	26.5	19.9	19.9	19.6	21.4

^a^By international data on cost per QALY gained

Since the Committee did not rank the rejected technologies, we assigned them a value of 26.5, the mean rank of the technologies rejected whatever their actual ranking is (19–34).

According to Table 2, four technologies were rejected by the Committee but ranked relatively high by the DCE: Gardasil and Zyban were ranked between second and fourth, and Apo-Go and Azilect were ranked 12^th^-14^th^. On the other hand, Eloxatin and Xeloda were accepted by the Committee (ranked 5^th^-6^th^), but were ranked relatively low (22^nd^-23^rd^) by the sample, and Crestor-Ezetrol (1^st^ choice of the Committee) was ranked 22^nd^-26^th^ by the sample. Growth hormone, an accepted technology ranked 16^th^ by the Committee, was ranked 22^nd^ by the sample. Finally, Plavix prescribed to stroke patients, was ranked 16^th^ by the Committee, but was the first choice of health policy-makers in the sample.

By definition, the mean Committee’s rank of the accepted technologies is 9.5, and that of the rejected ones is 26.5. In the sample of health policy-makers, the corresponding means are 14.8 and 19.3 respectively, indicating that, on the whole, the policy-makers found the accepted technologies less desirable, and the rejected ones – less undesirable. The means among the physicians and those who have ever been members of the Basket Committee in the sample were 16.9 vs. 19.2 and 16.1 vs. 18.8 respectively. The efficiency ranking presents a similar picture, though closer to the Committee’s ranking – the means are 14 and 21.4 respectively.

When the monthly cost threshold increased to 250 NIS (Table 3), four technologies – Plavix (stroke), Gardasil, Emend and Crestor Ezetrol - turned to be characterized by “no” instead of “yes” on the criteria “the technology should be financed publicly so that the poor can use it” (namely, their monthly cost is lower than 250 NIS – but higher than 125 NIS). Their rank dropped considerably: from 1^st^ to 7^th^, from 9^th^ to 23^rd^, from 25^th^ to 33^rd^ and from 18^th^ to 28^th^ respectively. Consequently, Lantus Levemir, a relatively cheap technology advanced from the 29^th^ to the 17^th^ place.

The increased threshold made the Committee’s accepted technologies more desirable by the sample of policy-makers.

Tables 4 and 5 present similar rankings, but for the 18 technologies accepted. In Table 4, the top four technologies accepted by the Committees were ranked 12-15^th^ by the sample. On the other hand, Plavix for stroke patients was ranked 17^th^ by the Committee but was ranked 1^st^ in the sample, and Mabthera – 11^th^ by the Committee and 1^nd^ by the sample. Velcade, ranked last (18^th^) by the Committee, was seen as more desirable (4^th^-5^th^) by the sample.

**Table 4:** The Committee’s and the sample ranking of technologies accepted by the 2006/7 Basket Committee (monthly cost threshold = 125 NIS)

Technology	Rank Basket Committee	Diagonal	Rank survey - Total	Rank survey - Physicians	Rank survey - Members of the Basket Committee	Rank efficiency^a^
Crestor Ezetrol	1	1	12	12	10	9
Atacand Ocsaar Diovan Olmetec	2	2	15	15	15	2
Lantus Levemir	3	3	15	15	15	4
Apidra Humalog Novorapid	4	4	15	15	15	7
Eloxatin	5	5	10	10	10	3
Xeloda	6	6	10	10	10	1
Herceptin	7	7	8	8	9	15
Hepsera	8	8	3	3	3	12
Viread	9	9	7	6	7	14
Prevnar	10	10	18	18	18	18
Mabthera	11	11	1	1	1	11
Keppra	12	12	13	13	14	5
Zyprexa	13	13	14	14	13	17
Exjade	14	14	5	4	4	13
Plavix (cardiac)	15	15	6	6	5	8
Growth hormon	16	16	9	9	8	10
Plavix (stroke)	17	17	1	1	1	6
Velcade	18	18	4	5	5	16

^a^By international data on cost per QALY gained

**Table 5:** The Committee’s and the sample ranking of technologies accepted by the 2006/7 Basket Committee (monthly cost threshold = 250 NIS)

Technology	Rank Basket Committee	Diagonal	Rank survey - Total	Rank survey - Physicians	Rank survey - Members of the Basket Committee	Rank efficiency^a^
Crestor Ezetrol	1	1	15	15	15	9
Atacand Ocsaar Diovan Olmetec	2	2	15	15	15	2
Lantus Levemir	3	3	12	12	10	4
Apidra Humalog Novorapid	4	4	15	15	15	7
Eloxatin	5	5	10	10	10	3
Xeloda	6	6	10	10	10	1
Herceptin	7	7	8	8	9	15
Hepsera	8	8	2	2	2	12
Viread	9	9	7	5	7	14
Prevnar	10	10	18	18	18	18
Mabthera	11	11	1	1	1	11
Keppra	12	12	13	13	14	5
Zyprexa	13	13	14	14	13	17
Exjade	14	14	4	3	3	13
Plavix (cardiac)	15	15	5	5	4	8
Growth hormon	16	16	9	9	8	10
Plavix (stroke)	17	17	5	5	4	6
Velcade	18	18	3	4	4	16

^a^By international data on cost per QALY gained

When the monthly cost threshold increased to 250 NIS (Table 5), Plavix (stroke) and Crestor Ezetrol, the two accepted technologies which cost below 250 NIS (but above 125 NID), lost some of their desirability and their ranking dropped from 12^th^ to 15^th^, and from 1^st^ to 5^th^ respectively.

Tables 6 and 7 present summary measures – correlations - of the joint distributions of the sample’s rankings, the Committee’s ranking and the efficiency ranking of all and accepted technologies respectively, and for the two monthly cost thresholds.

**Table 6:** Correlations among the different rankings: all technologies, monthly cost threshold = 125 and 250 NIS

	Rank Basket Committee^a^	Rank survey - Total	Rank survey - Physicians	Rank survey - Members of the Basket Committee	Rank efficiency
Monthly cost threshold = 125 NIS					
Correlation with the Committee’s ranking - All technologies (survey: 1–34, Committee: NO = 26.5)	1.000	0.035	0.036	0.096	0.450^a^
Correlation with the Efficiency ranking - All technologies (survey: 1–34, Committee: NO = 26.5)	0.450^a^	0.067	0.040	0.176	1.000
Monthly cost threshold = 250 NIS					
Correlation with the Committee’s ranking - All technologies (survey: 1–34, Committee: NO = 26.5)	1.000	0.101	0.106	0.150	0.450^a^
Correlation with the Efficiency ranking - All technologies (survey: 1–34, Committee: NO = 26.5)	0.450^a^	0.136	0.132	0.241	1.000

^a^Significant at 0.05

**Table 7:** Correlations among the different rankings: accepted technologies, monthly cost threshold = 125 and 250 NIS

	Rank Basket Committee^a^	Rank survey - Total	Rank survey - Physicians	Rank survey - Members of the Basket Committee	Rank efficiency
Monthly cost threshold = 125 NIS					
Correlation with the Committee’s ranking - Accepted technologies (survey:1–18)	1.000	−0.541^a^	−0.528^a^	−0.529^a^	0.434^a^
Correlation with the Efficiency ranking - Accepted technologies (survey: 1–18)	0.434^b^	−0.146	−0.149	−0.150	1.000
Monthly cost threshold = 250 NIS					
Correlation with the Committee’s ranking - Accepted technologies (survey:1–18)	1.000	−0.530^a^	−0.513^a^	−0.537^a^	0.434^b^
Correlation with the Efficiency ranking - Accepted technologies (survey: 1–18)	0.434^b^	−0.169	−0.179	−0.142	1.000

^a^Significant at 0.05

^b^significant at 0.10

Table 6 presents the correlations for all technologies. Rejected technologies were assigned the mean of 19–34, 26.5. For the 125 and 250 NIS thresholds, the sample’s and the Committee’s rankings are uncorrelated. The correlation between the Committee’s and the efficiency rankings is relatively high and significant (0.45).

Among accepted technologies (Table 7), for both cost thresholds, the sample’s and the Committee’s rankings are relatively highly and significantly negatively correlated. The efficiency ranking is uncorrelated with the sample’s rankings, and correlates (marginally significant) positively with the Committee’s ranking.

**Fig. 1:**
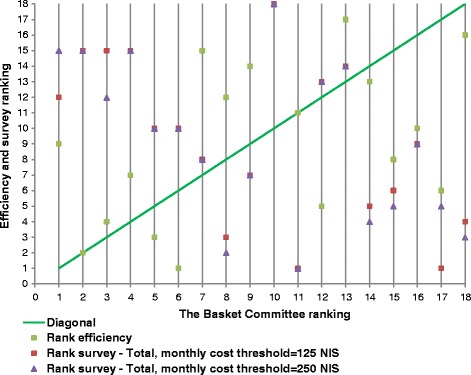
The Committee’s, survey and efficiency rankings of the accepted technologies

Figure 1 portrays the fit of the Committee’s, the total sample’s, and the efficiency rankings graphically. The Figure indicates that, with the two cost thresholds, the lack of fit concentrates in the extremes: the four top and the bottom two technologies of the Committee’s ranking are ranked almost inversely by the sample.

## Discussion

The prioritization of new technologies submitted for public finance, expected from the Basket Committee, is the most dramatic manifestation of the tension between equity and efficiency considerations in health policy.

We used a recent DCE to predict - based on the stated preferences of Israeli health policy-makers - the desirability of 18 technologies which were accepted by the 2006/7 Basket Committee and 16 which were not.

The results show that, in general, the ranking resulted from the DCE does not agree with the Committee’s inclusion decisions and its ranking. The correlation between the two is null for the entire set of technologies, and negative for the accepted technologies. In particular, the disagreement is pronounced in the two extremes: top technologies accepted by the Committee are ranked 15^th^ (out of 18) by the sample, and the two last technologies ranked 17^th^-18^th^ by the Committee were ranked 4^th^-5^th^ by the sample.

Interestingly, the Committee’s inclusion decisions (and to a lesser extent - the ranking of the accepted technologies) are more related to efficiency considerations than to the ranking derived from the preferences of Israeli health policy-makers. As was mentioned earlier, the Israeli health policy-makers tend, at the declarative level, to balance equity and efficiency considerations. It seems that, in general, the members of the Basket Committee tend to prefer efficiency considerations more than health policy-makers in general.

Several explanations are suggested. First, the survey’s ranking is based on *stated* preferences while that of the Committee reflects *revealed* preferences. Real-life decisions are complex and involve many dimensions. Real-life prioritizations are based on additional criteria which were not included in the seven presented to the respondents. Furthermore, these criteria might interact in a way that the additive utility construct, which is the basis for DCE, cannot accommodate.

Second, the finding that the Committee’s ranking agrees more with the efficiency ranking might be a result of the real budget constraint under which the Committee prioritizes the technologies. Facing a budget constraint, one of the major considerations of the Committee is the budget impact of the technology under consideration (namely, what share of the budget would the technology use if accepted). Since cheap technologies are more likely than expensive ones to have both lower budget impact and to be cost-effective (i.e., to rank high on the efficiency ranking), the Committee’s inclusion decision and its ranking are expected to be more correlated with the efficiency ranking than with the survey’s, even if the chief efficiency consideration - cost per QALY criterion - is assigned a low weight or even ignored.

A confirmation of these two explanations stems from the comparison of the weights – and hence the ranking – attached to the criteria by health policy-makers who have ever served on a Basket Committee with those of health policy-makers who have not. As in the case of the actual Committee’s prioritization, those who have had the experience of “real” deliberations, assign a higher weight to efficiency considerations at the expense of equity ones.

The third possible explanation is related to the fact that some of the Committee’s members are “public nominations” rather than policy-makers. However, if the preferences of policy-makers in the Committee are similar to those surveyed, that would mean that the “public nominations” show a higher tendency towards efficiency, which is hardly believable.

Finally, the time gap between the Committee’s decisions and ranking and that derived from the survey is almost 10 years. Social norms and values might have changed over the years, leading to differences in the preferences of the Israeli health policy-makers.

Clearly, the task faced by the Committee is not an easy one. However, the existing “guidelines” and “instructions” (issued by the Ministry of Health) for the work of the Committee relate more to the organizational aspects and to the principles to be discussed, rather than trying to provide a more structured framework to the prioritization process, namely, which criteria to be used and what are their relative weights. Consequently, the Committee’s decision process is not transparent, hard to be evaluated, unclear and might be inconsistent.

More and more researchers comment on the work of the Israeli Committee and on its decisions [7–12]. Golan and Hansen [12] even propose a decision-support tool based on the principle of “value for money”. The present findings point to the need of health policy-makers in Israel to align and coordinate their decision processes with respect to the equity and efficiency criteria for prioritization and their weights.

## Conclusions

The findings presented above indicate that the preferences of the members of the Basket Committee regarding the efficiency-equity tradeoff do not match those of health policy makers. It seems that in real life prioritization facing a budget constraint, health policy makers turn toward efficiency considerations. In order for Israeli health policy to be consistent and well coordinated across policy-makers in various functions , some discussions and exchanges are needed in order to arrive at a common set of preferences with respect to equity and efficiency considerations.
